# Resveratrol Attenuates Obesity by Inducing GDF15 Expression via the p38‐ATF3 Signaling Pathway

**DOI:** 10.1002/fsn3.72052

**Published:** 2026-06-28

**Authors:** Liufeng Mao, Yunliang Hou, Weifang Liu, Jianbin Chen, Wanli Hu, Wengong Jiang, Tao Nie

**Affiliations:** ^1^ Central Laboratory The First Affiliated Hospital, Guangdong Pharmaceutical University Guangzhou China; ^2^ Neurological Research Institute of Integrated Traditional Chinese and Western Medicine The First Affiliated Hospital, Guangdong Pharmaceutical University Guangzhou China; ^3^ Nephrology Department The First Affiliated Hospital, Guangdong Pharmaceutical University Guangzhou China; ^4^ School of Basic Medicine Hubei University of Arts and Science Xiangyang China

**Keywords:** ATF3, ERK signaling, GDF15, obesity, resveratrol

## Abstract

Resveratrol, a natural polyphenol, has been widely studied for its potential health benefits in obesity treatment, but its molecular mechanism remains elusive. This study investigates the molecular mechanisms by which resveratrol promotes the expression and secretion of Growth Differentiation Factor 15 (GDF15), a key regulator of energy homeostasis. Using both murine embryonic fibroblasts (MEFs) and human HepG2 cells, resveratrol dose‐dependently upregulates *Gdf15* mRNA and protein expression, as well as increases GDF15 secretion. Transcriptome analysis and subsequent validation experiments revealed that Activating Transcription Factor 3 (ATF3) mediates the effect of resveratrol on GDF15 expression. Additionally, resveratrol‐treated mice exhibited resistance to high‐fat diet‐induced obesity and increased plasma GDF15 levels. In vivo studies in mice further confirmed that resveratrol treatment elevates GDF15 levels in liver, kidney, and colon tissues, and enhances downstream ERK signaling in the brain. Collectively, these findings suggest that GDF15 plays a critical role in mediating the anti‐obesity effects of resveratrol.

## Graphical abstract

Resveratrol dose‐dependently upregulates GDF15 expression in murine and human cells via the p38‐ATF3 signaling axis. In high‐fat diet fed mice, resveratrol elevates systemic and tissue GDF15 levels, thereby reducing food intake and weight gain and conferring resistance to diet‐induced obesity. This study identifies a novel p38‐ATF3‐GDF15 axis mediating resveratrol's anti‐obesity effects.

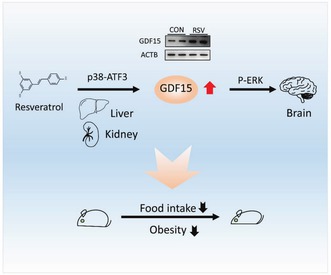

## 1 Introduction

Obesity and its associated metabolic disorders have reached epidemic proportions worldwide (Ng et al. 2014). Lifestyle interventions—chiefly caloric restriction and increased physical activity—remain the first‐line strategy for weight management, yet their long‐term success is limited by poor adherence and physiological counter‐regulation.

Resveratrol (3,4,5‐trihydroxystilbene), a polyphenolic phytoalexin abundant in mulberry, Japanese knotweed, peanuts, red grapes and wine (Bartusik‐Aebisher et al. 2019; Baur and Sinclair 2006), has consistently demonstrated protective effects against diet‐induced obesity, hepatic steatosis, chronic inflammation, and dyslipidemia in pre‐clinical models (Balakina et al. 2025; Bartusik‐Aebisher et al. 2019; Chen et al. 2012; Ji et al. 2015; Qureshi et al. 2012; Y. Zhang et al. 2015). Beyond its peripheral metabolic actions, resveratrol also appears to modulate food intake, although its effects are context‐dependent and not consistently observed across all studies. In a seasonal non‐human primate model (gray mouse lemurs, *Microcebus murinus* ), resveratrol supplementation reduced spontaneous food intake and attenuated winter‐weight gain, although circulating levels of GLP‐1, PP and PYY remained unchanged (Dal‐Pan et al. 2010). Conversely, C57BL/6J mice receiving resveratrol exhibited a modest rise in serum GLP‐1; yet this increase was not causally linked to weight loss but rather to improved glycaemic control (Dao et al. 2011). In addition, resveratrol suppresses cumulative food intake in C57BL/6J mice by markedly down‐regulating hypothalamic expression of the orexigenic neuropeptides NPY and AgRP (Kim et al. 2010). In rat models of early‐weaning (EW)‐induced obesity, resveratrol normalized food intake and prevented excess weight gain and visceral adiposity. It restored hypothalamic leptin sensitivity by increasing JAK2 and STAT3 phosphorylation and suppressing NPY expression, thereby reversing EW‐induced central leptin resistance (Franco et al. 2014, 2013). Despite these promising findings, the precise molecular mechanisms underlying resveratrol's anti‐obesity effects remain incompletely understood, limiting its translational potential as a therapeutic agent.

In recent years, Growth Differentiation Factor 15 (GDF15) has emerged as a critical regulator of energy homeostasis and body weight (Breit et al. 2023; J. Zhang et al. 2024). GDF15 is a member of the transforming growth factor‐beta (TGF‐β) superfamily and is expressed in various tissues, including the liver, kidney, and colon. Under physiological conditions, GDF15 levels are relatively low, but they are markedly elevated in response to cellular stress, such as oxidative stress, inflammation, and mitochondrial dysfunction (D. Wang et al. 2021). GDF15 exerts its effects by binding to the glial cell line‐derived neurotrophic factor (GDNF) family receptor α‐like (GFRAL) in the hindbrain, leading to activation of ERK signaling pathway and subsequent suppression of appetite (Mullican et al. 2017; Rochette et al. 2020). Elevated levels of GDF15 have been associated with reduced food intake, improved insulin sensitivity, and protection against obesity in both animal models and humans (Hale and Véniant 2021; J. Zhang et al. 2024). The regulation of GDF15 expression is complex and involves multiple signaling pathways and transcription factors (Aguilar‐Recarte et al. 2022; M. H. Yang et al. 2014). One such transcription factor is Activating Transcription Factor 3 (ATF3), a member of the ATF/CREB family of transcription factors that plays a key role in cellular stress responses (Lu et al. 2006). ATF3 is rapidly induced in response to various stressors, including endoplasmic reticulum (ER) stress and oxidative stress, and regulates GDF15 expression by directly binding in its promoter region (Baek 2004).

The primary objective of this study was to investigate the molecular mechanisms by which resveratrol promotes GDF15 expression and secretion and to determine whether GDF15 mediates the anti‐obesity effects of resveratrol. This study provides novel insights into the mechanisms underlying resveratrol's metabolic benefits on obesity and its related metabolic disorders.

## 2 Materials and Methods

### 2.1 Cell Culture and Treatments

Murine embryonic fibroblasts (MEFs) and human HepG2 hepatoma cells were cultured in Dulbecco's Modified Eagle Medium (DMEM) supplemented with 10% fetal bovine serum (FBS) and 1% penicillin–streptomycin at 37°C in a 5% CO_2_ atmosphere. Cells were treated with resveratrol (Targetmol, T1558) at the indicated concentrations. An equivalent volume of dimethyl sulfoxide (DMSO) was used as the vehicle control in all experiments. For pathway inhibition studies, cells were treated with the p38 specific inhibitor SB856553 (Targetmol, T2277).

### 2.2 RNA Sequencing and Transcriptome Analysis

Total RNA was extracted from MEF cells treated with or without 10 μM resveratrol for 24 h using TRIzol reagent (Invitrogen, 15,596,026). RNA integrity was verified, and sequencing libraries were prepared and sequenced by Shanghai Majorbio Bio‐pharm Technology Co. Ltd. Differential gene expression analysis was performed using the EdgeR package on the Majorbio I‐Sanger Cloud Platform. Genes with a fold change > 2 and a false discovery rate (FDR) < 0.05 were considered significantly differentially expressed.

### 2.3 RNA Extraction and Quantitative Real‐Time PCR (qPCR)

Total RNA from cells and tissues was reverse‐transcribed into cDNA using Superscript III Reverse Transcriptase (Invitrogen, 18,080,085). Quantitative PCR was performed using SYBR Premix Ex Taq (Genstar，A311‐10) on a BioRad Real‐Time PCR System. The 18S ribosomal RNA gene was used as an internal control for normalization. The relative mRNA expression levels were calculated using the 2^(−ΔΔCt) method.

### 2.4 Enzyme‐Linked Immunosorbent Assay (ELISA)

The concentration of GDF15 in cell culture supernatants and mouse serum was quantified using a commercial mouse and human GDF15 ELISA kit (R&D Systems, MGD150 and DGD150) according to the manufacturer's instructions.

### 2.5 Western Blot Analysis

Cells and homogenized tissues were lysed in RIPA buffer supplemented with protease and phosphatase inhibitors. Protein concentrations were determined using a BCA assay. Equal amounts of protein were separated by SDS‐PAGE, transferred to PVDF membranes, and probed with specific primary antibodies against GDF15 (Abclonal, A0185), ATF3 (Abclonal, A1852), P38MAPK (Abclonal, A4771), Phospho‐P38MAPK (Abclonal, AP1372), phospho‐ERK (CST, 4370), total ERK (CST, 4695), GAPDH (CST, 5174 T) and beta‐actin (ACTB) (Abclonal, AC026). After incubation with appropriate HRP‐conjugated secondary antibodies, protein bands were visualized using an enhanced chemiluminescence substrate and quantified with ImageJ software.

### 2.6 Small Interfering RNA (siRNA) Knockdown

MEF cells were transfected with either a non‐targeting control siRNA or siRNA targeting *Atf3* using Lipofectamine 3000 transfection reagent (Invitrogen, L3000150). After 48 h of transfection, cells were treated with resveratrol for the indicated times before harvesting for qPCR analysis.

### 2.7 In Vivo Studies

All animal experiments were conducted in accordance with the Guide for the Care and Use of Laboratory Animals and were approved by the Animal Care and Use Committee of Guangdong Pharmaceutical University. Ten‐week‐old male C57BL/6J mice were randomly divided into two groups. The treatment group received resveratrol (250 mg/kg body weight) via daily oral gavage, while the control group received an equivalent volume of vehicle. Mice were fed either a standard chow diet or a high‐fat diet (60% kcal from fat, Research Diets, D12492) for 3 weeks. Body weight or food intake were recorded weekly or daily. At the end of the study, blood, liver, kidney, colon, and brain tissues were collected for further analysis.

### 2.8 Statistical Analysis

Data are presented as the mean ± standard error of the mean (SEM). Statistical comparisons between two groups were analyzed using an unpaired, two‐tailed Student's *t*‐test. A *p*‐value of less than 0.05 was considered statistically significant.

## 3 Results

### 3.1 Resveratrol Promotes Expression and Secretion of GDF15 in Murine and Human Cells

**FIGURE 1:**
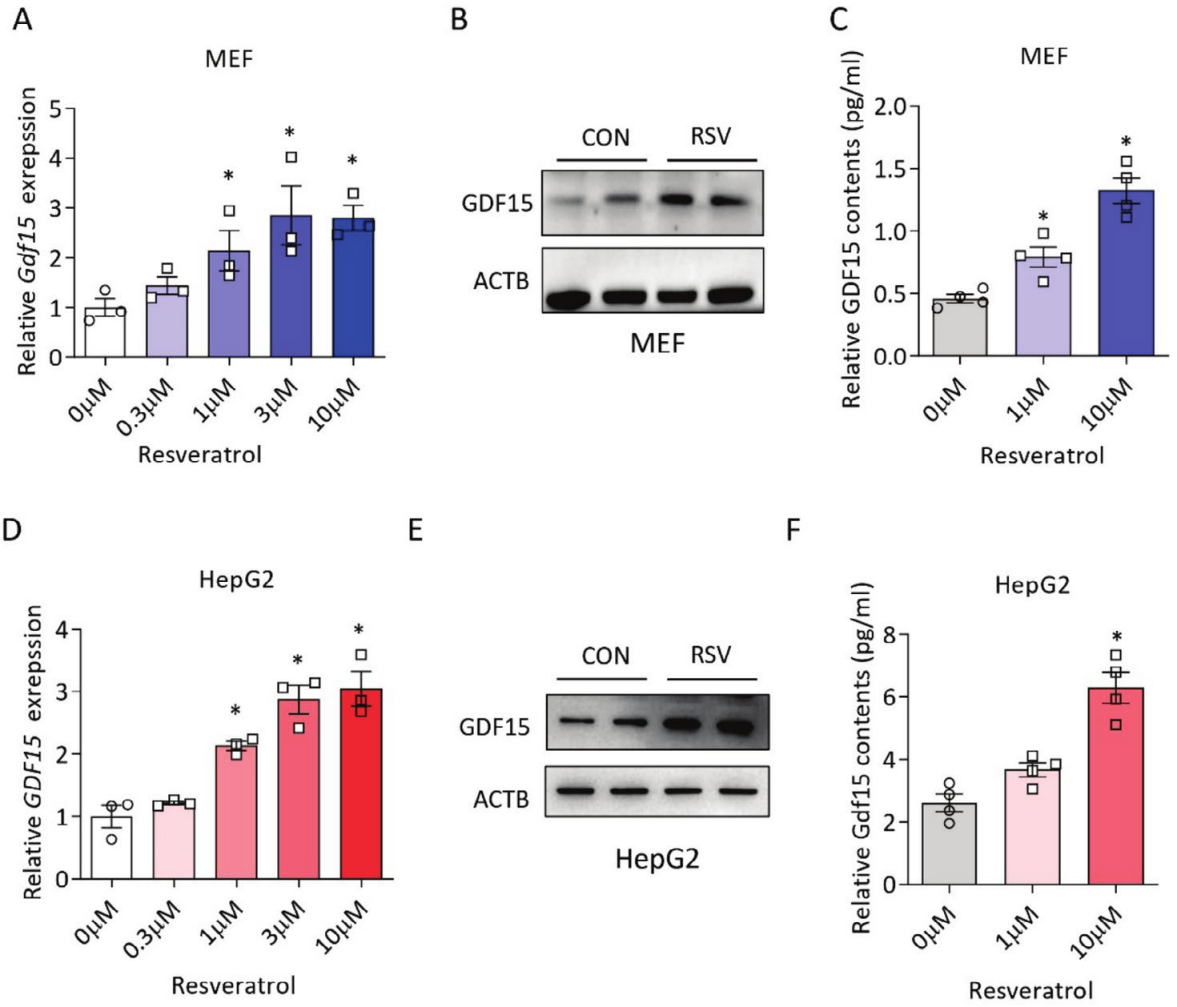
Resveratrol promotes GDF15 expression and secretion in murine and human cells. (A) Relative *Gdf15* mRNA levels in MEF cells treated with increasing doses of resveratrol for 24 h, as determined by qPCR. (B) Intracellular GDF15 protein levels in MEF cells after resveratrol treatment (10 μM, 24 h), assessed by Western blot. (C) Secreted GDF15 protein in culture media of MEF cells treated with resveratrol (10 μM), measured by ELISA. (D, E) GDF15 mRNA (D) and protein (E) levels in *HepG2* cells following resveratrol treatment (10 μM). (F) Secreted GDF15 protein in culture media of *HepG2* cells treated with resveratrol (10 μM), measured by ELISA. Data are presented as mean ± SEM; *p < 0.05.

To investigate the effect of resveratrol on the expression and secretion of GDF15, we utilized murine embryonic fibroblast (MEF) cells and human HepG2 cells. MEF cells serve as a well‐established cellular platform and are widely employed as powerful tools for investigating molecular mechanisms of protein function and cellular signaling, including the regulation of GDF15 expression. (Coll et al. 2020; Howerton et al. 2008; Kang et al. 2025). As illustrated in Figure 1A, resveratrol (RSV) treatment resulted in a dose‐dependent increase in *Gdf15* mRNA expression in MEF cells. Correspondingly, Western blot analysis confirmed elevated intracellular GDF15 protein levels (Figure 1B), and ELISA assays revealed a significant increase in GDF15 secretion into the culture medium (Figure 1C). Similar enhancements in GDF15 expression and secretion were observed in human HepG2 cells, suggesting that the regulatory effect of resveratrol on GDF15 may be conserved across species, which requires further verification in more cell models (Figure 1D–E). Collectively, these findings demonstrate that resveratrol effectively upregulates both the expression and secretion of GDF15 in vitro.

### 3.2 ATF3 Mediates the Effect of Resveratrol on GDF15 Expression

**FIGURE 2:**
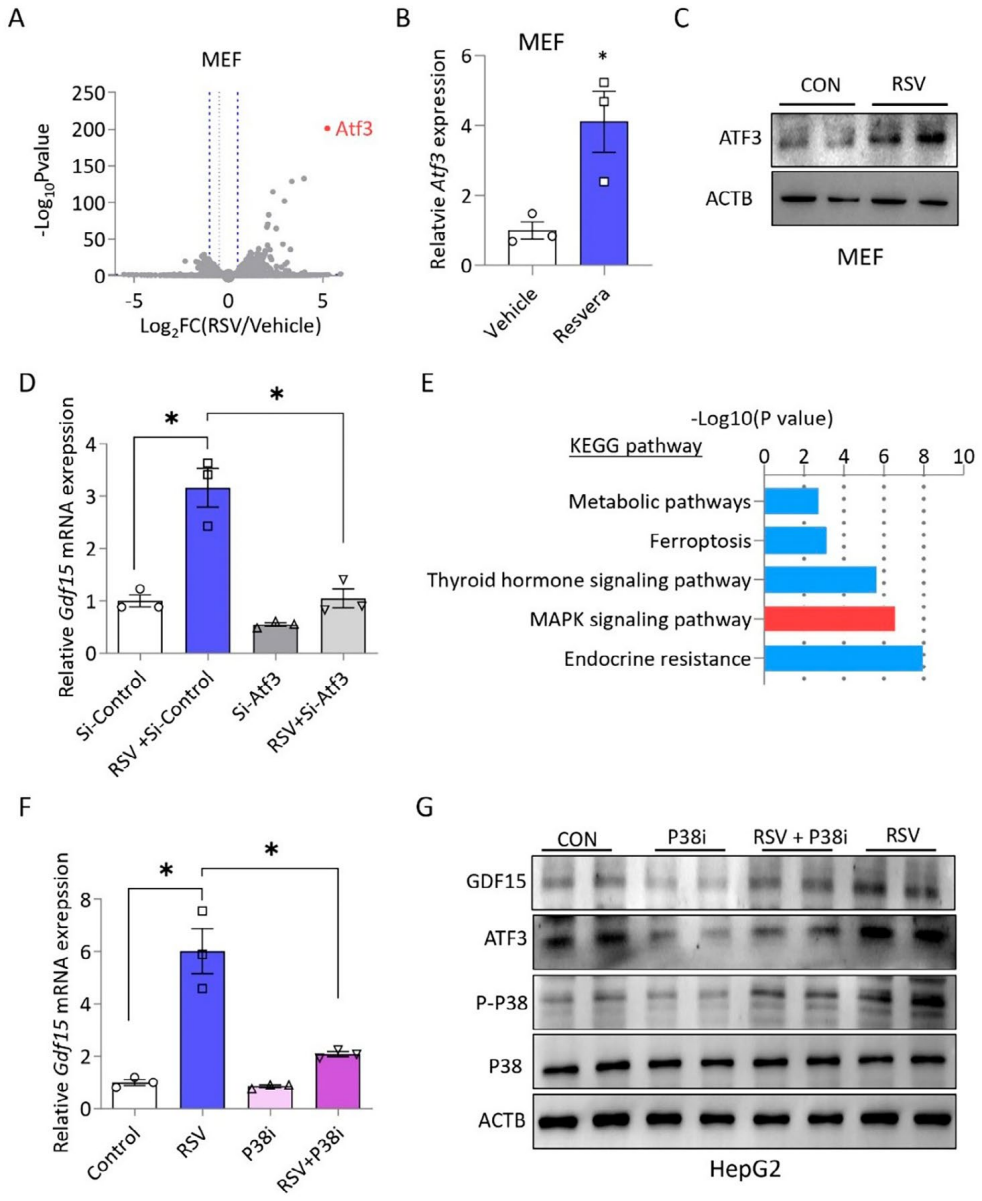
The p38‐ATF3 axis mediates resveratrol‐induced GDF15 expression. (A) Volcano plot from RNA‐seq analysis of MEF cells treated with resveratrol (10 μM), highlighting *Atf3* as a significantly upregulated transcript (A). ATF3 mRNA (B) and protein (C) expression in resveratrol‐treated MEF cells. *Gdf15* mRNA levels in MEF cells transfected with control or *Atf3*‐targeting siRNA, followed by resveratrol treatment (D). KEGG pathway enrichment analysis of differentially expressed genes from RNA‐seq data (E). *Gdf15* mRNA expression in MEF cells treated with a p38 inhibitor (SB203580, 10 μM) (F). Levels of ATF3, GDF15, p38, and p‐p38 proteins in HepG2 cells treated with resveratrol, a p38 inhibitor, or a combination of both (G). Data are mean ± SEM; *p < 0.05.

To further explore the molecular mechanism by which resveratrol regulates *Gdf15* expression, we performed transcriptomic RNA‐seq analysis. Volcano plot analysis revealed a marked upregulation of *Atf3* expression in resveratrol‐treated MEF cells (Figure 2A). Subsequent qPCR and Western blot analyses validated that resveratrol significantly increased both ATF3 mRNA and protein levels (Figure 2B,C). Importantly, siRNA‐mediated knockdown of *Atf3*, which achieved approximately 50% knockdown efficiency in MEF cells (Figure S1), substantially attenuated the resveratrol‐induced upregulation of *Gdf15* expression (Figure 2D). KEGG pathway analysis implicated the MAPK signaling pathway in resveratrol's mechanism of action (Figure 2E). Given previous reports linking the p38 MAPK pathway to *Atf3* regulation (Lu et al. 2006), we employed a p38 inhibitor, which effectively suppressed resveratrol‐induced *Gdf15* expression (Figure 2F). Moreover, resveratrol enhanced the phosphorylation of the p38 signaling pathway, while treatment with a p38 inhibitor not only reduced ATF3 expression but also diminished the resveratrol‐induced upregulation of both ATF3 and GDF15 in HepG2 cells (Figures 2G and S2). Together, these results indicate that the p38–ATF3 signaling axis mediates the stimulatory effect of resveratrol on GDF15 expression.

### 3.3 Resveratrol Increases GDF15 Expression and Confers Resistance to Obesity in Vivo

**FIGURE 3:**
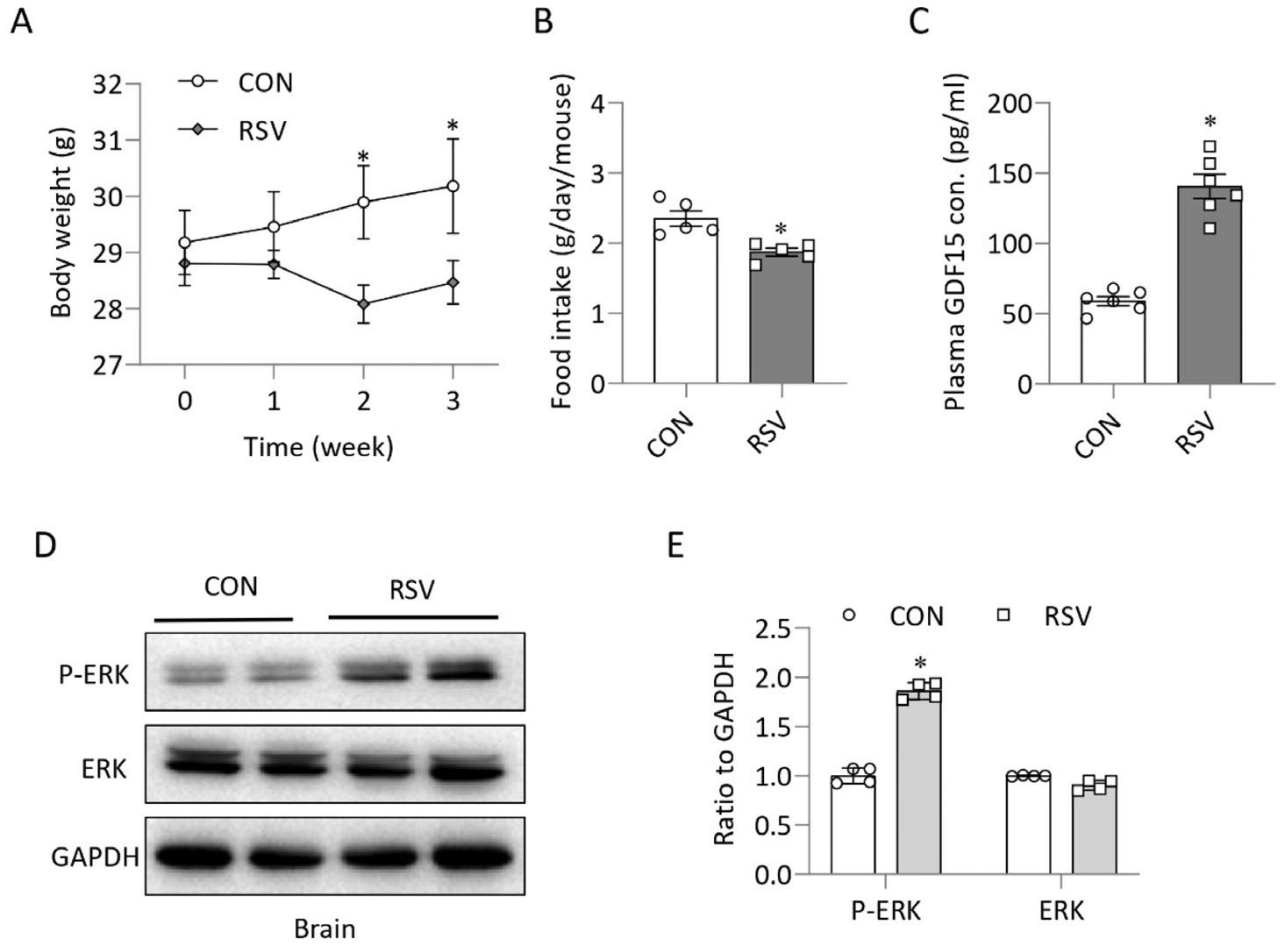
Resveratrol treatment increases circulating GDF15 level and reduces weight gain in mice fed a high‐fat diet. (A) Body weight of HFD‐fed mice treated with or without resveratrol for 3 weeks. (B) Average weekly food intake in HFD‐fed mice with or without resveratrol treatment. (C) Serum GDF15 levels measured by ELISA. (D, E) Representative Western blot and quantification of phosphorylated ERK (p‐ERK) and total ERK in brain tissues. Data are mean ± SEM; *p < 0.05.

We next evaluated the physiological relevance of resveratrol treatment in vivo. Male C57BL/6J mice received standard chow or 60% HFD with or without RSV (250 mg/kg, oral gavage) for 3 weeks. In mice maintained on a standard chow diet, resveratrol administration led to reduced food intake and elevated serum GDF15 levels (Figure S3A,B). When challenged with a high‐fat diet (HFD), resveratrol‐treated mice exhibited attenuated weight gain and suppressed food intake compared with controls (Figure 3A,B). Consistent with these metabolic improvements, serum GDF15 concentrations were significantly higher in resveratrol‐treated mice (Figure 3C). Furthermore, enhanced activation of the ERK signaling pathway—a downstream target of GDF15—was observed in the brain of resveratrol‐treated mice (Figure 3D), aligning with the elevated systemic GDF15 levels.

**FIGURE 4:**
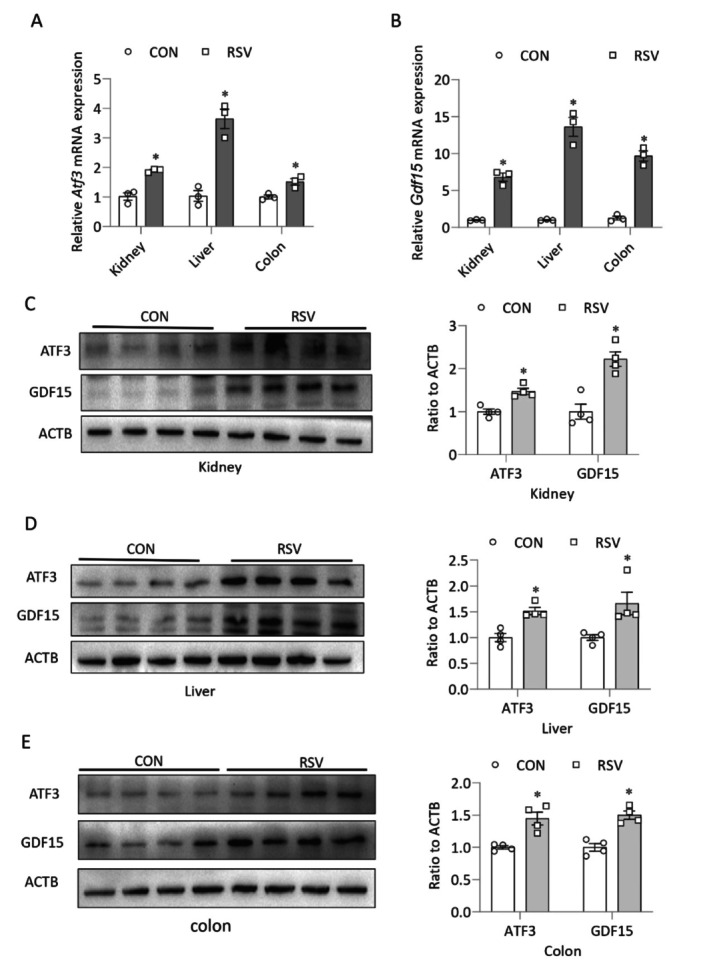
Tissue induction of ATF3 and GDF15 by resveratrol in vivo. (A, B) Relative mRNA expression of *Atf3* (A) and *Gdf15* (B) in liver, kidney, and colon tissues from resveratrol‐treated mice. (C–E) Representative Western blots and quantification of GDF15 protein expression in kidney (C), liver (D), and colon (E) tissues. Data are mean ± SEM; *p < 0.05.

**FIGURE 5:**
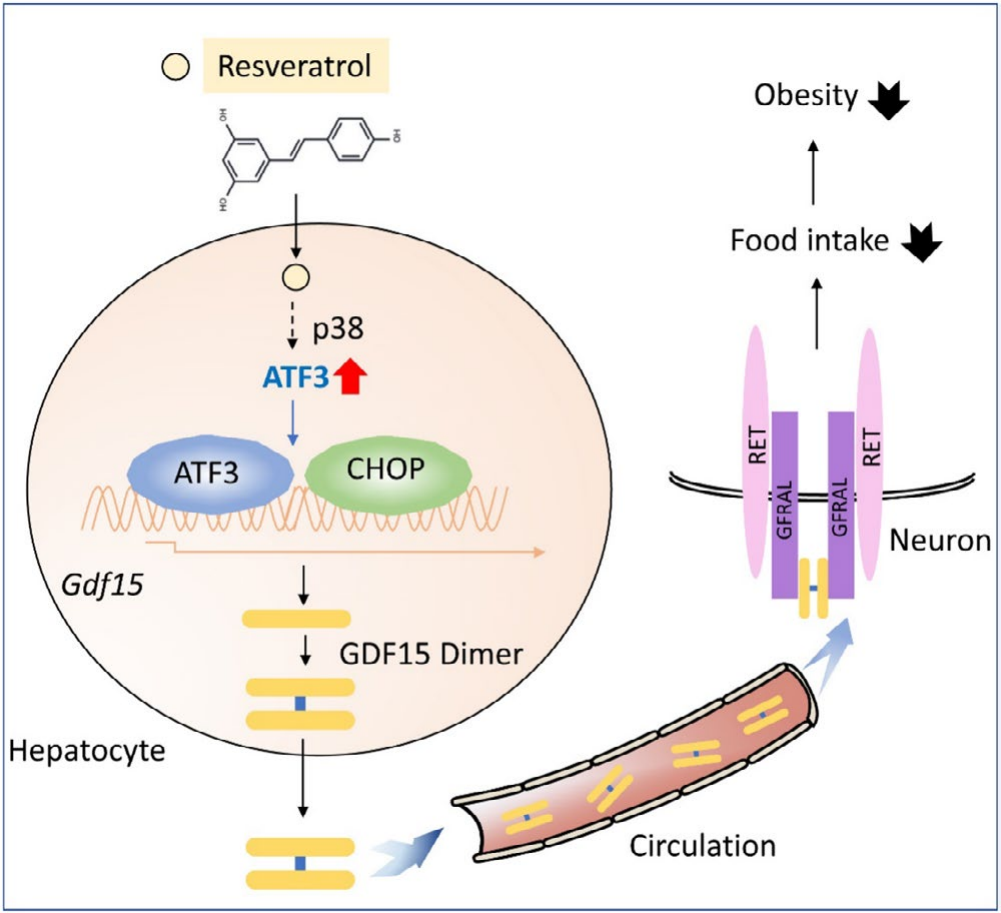
Proposed model for resveratrol‐induced body weight reduction via the GDF15 pathway. Schematic illustration summarizing the proposed mechanism: Resveratrol activates the p38 MAPK signaling pathway, leading to upregulation of ATF3. This transcription factor then promotes GDF15 expression and secretion in peripheral tissues. The elevated circulating GDF15 acts on the brain, potentially through activation of the ERK pathway in the hypothalamus, resulting in reduced food intake and subsequent protection against diet‐induced obesity.

Tissue‐specific analysis revealed that resveratrol treatment increased the mRNA expression of both *Atf3* and *Gdf15* in the liver, kidney, and colon (Figure 4A,B). Western blot analysis further confirmed elevated GDF15 protein expression in these tissues (Figure 4C–E). In summary, our data support a model in which GDF15, likely acting through the brain ERK pathway, contributes to the body weight‐reducing effects of resveratrol (Figure 5).

## 4 Discussion

Our study provides the first comprehensive evidence that resveratrol induces GDF15 expression via activation of the p38–ATF3 signaling axis, contributing to its anti‐obesity effects in both cellular and animal models. Our findings demonstrate that resveratrol dose‐dependently upregulates GDF15 mRNA and protein expression in both MEF and human HepG2 cells. The upregulation of GDF15 by resveratrol in human cells is particularly noteworthy, as it suggests that this mechanism may be conserved across species and could have translational relevance for human health. This is consistent with previous reports showing that phytochemicals can modulate stress‐responsive pathways to confer metabolic benefits (Li et al. 2023; L. Wang et al. 2024).

A key finding of this study is the identification of ATF3 as a critical mediator of resveratrol's effects on GDF15 expression. ATF3 is a stress‐inducible transcription factor that integrates signals from multiple MAPK pathways, including p38, JNK, and ERK (Lu et al. 2006). Our data show that resveratrol rapidly induces ATF3 expression in a p38‐dependent manner, and that silencing *Atf3* abolishes GDF15 upregulation. This is in line with previous studies demonstrating that ATF3 can bind to the promoter region of GDF15 and enhance its transcription (Piyanuch et al. 2007). Thus, our findings establish a clear mechanistic link between p38 activation, ATF3 induction, and GDF15 expression in the context of resveratrol treatment.

In vivo, resveratrol administration significantly reduced body weight gain and food intake in HFD‐fed mice, effects that were paralleled by elevated serum GDF15 levels and enhanced hypothalamic ERK phosphorylation. These results align with earlier studies showing that central GDF15–GFRAL signaling leads to ERK activation in the area postrema and nucleus tractus solitarius, ultimately suppressing appetite (Mullican et al. 2017; Suriben et al. 2020). Moreover, tissue‐specific upregulation of GDF15 and ATF3 in liver, kidney, and colon suggests that resveratrol may act through multiple organ systems to modulate systemic metabolism. This is consistent with previous findings showing that resveratrol can influence gut microbiota composition, hepatic lipid metabolism, and renal inflammatory responses (Das et al. 2025; Gu et al. 2025; M. Wang et al. 2020).

Consistent with previous preclinical evidence, our study confirms that resveratrol at a dose of 250 mg/kg/day is non‐toxic to the liver and kidney. Preclinical studies have demonstrated that 250 mg/kg/day resveratrol reduces serum alanine transaminase (ALT) levels, hepatic lipid accumulation, and inflammatory infiltration in mice with nonalcoholic steatohepatitis (NASH), while preserving core hepatic metabolic functions without inducing hepatocellular necrosis or fibrosis (Ji et al. 2015; C. Yang et al. 2019). For the kidney, 400 mg/kg/day resveratrol decreases blood urea nitrogen (BUN), microalbuminuria, and glomerular volume, inhibits renal inflammation and lipid accumulation, and maintains intact glomerular and tubular structure (Zhou et al. 2016). Additionally, resveratrol activates adaptive metabolic responses to reduce oxidative stress and improve insulin sensitivity, rather than to triggering toxic stress (Shabani et al. 2024). Toxicokinetic studies further validate that resveratrol doses up to 1250 (rats) and 2500 (mice) mg/kg do not induce overt toxicity, with a no‐observed‐adverse‐effect level (NOAEL) far exceeding our tested dose (Mutlu et al. 2020). Collectively, these findings confirm the safety of resveratrol at the administered dose, supporting that the observed upregulation of GDF15 and ATF3 reflects protective metabolic remodeling rather than the organ dysfunction.

Importantly, the anti‐obesity effects of resveratrol observed in this study are consistent with a growing body of literature demonstrating its role in promoting adipocyte thermogenesis, enhancing fatty acid oxidation, and inhibiting adipogenesis (Das et al. 2025; Terzo et al. 2024). For instance, resveratrol has been shown to activate brown adipose tissue and induce white adipose tissue browning via SIRT1/PGC‐1α‐dependent pathways (Pan et al. 2019). While these effects are complementary to our findings, the GDF15‐mediated appetite suppression represents a distinct and previously underappreciated mechanism by which resveratrol regulates energy balance.

## 5 Conclusion

While this study provides important insights into the mechanisms underlying resveratrol's anti‐obesity effects, it also has some limitations. First, the study primarily used in vitro and animal models, and further research is needed to confirm these findings in humans. Clinical trials are needed to evaluate the effects of resveratrol on GDF15 levels and metabolic health in humans. Second, the study focused on the role of ATF3 in mediating resveratrol's effects on GDF15 expression, but other transcription factors and signaling pathways may also be involved.

In conclusion, this study identifies a p38–ATF3–GDF15 signaling axis through which resveratrol exerts its anti‐obesity effects. These findings not only deepen our understanding of resveratrol's metabolic actions but also highlight GDF15 as a promising therapeutic target for obesity and related metabolic disorders.

## Author Contributions

**Yunliang Hou:** data curation, methodology, formal analysis, software, validation, writing – original draft. **Weifang Liu:** methodology, data curation. **Wengong Jiang:** writing – review and editing, formal analysis, supervision, investigation. **Liufeng Mao:** data curation, formal analysis, investigation, methodology, software, supervision, validation, writing – original draft. **Jianbin Chen:** methodology, data curation. **Wanli Hu:** methodology, data curation. **Tao Nie:** conceptualization, project administration, writing – review and editing, supervision.

## Funding

This study was supported, in part, by Key Project of Department of Education of Guangdong Provincial (2024ZDX2081, 2021JDA029), the Natural Science Foundation of Guangdong Province (2026A1515011102), Guangzhou Science and Technology Plan Project (2025A03J3666), Natural Science Joint Foundation of Hubei Province (2023AFD038), Innovation and Entrepreneurship Training Program for College Students (S202510519056), and Initial Funding of Hubei University of Arts and Science (2059200).

## Conflicts of Interest

The authors declare no conflicts of interest.

## Supporting information


**Figure S1:** Validation of Atf3 knockdown in MEF cells.
QPCR analysis demonstrates that transfection with Atf3‐specific siRNA effectively reduces endogenous Atf3 mRNA expression. Data are mean ± SEM; *p < 0.05.
**Figure S2:** Quantification of protein expression in HepG2 cells.
Quantification of ATF3 (A) and GDF15 (B) protein expression in HepG2 cells. Data are mean ± SEM; *p < 0.05.
**Figure S3:** Resveratrol effects in mice on standard chow diet.
(A) Average daily food intake in mice fed standard chow with or without resveratrol for 1 week. (B) Serum GDF15 levels after resveratrol treatment. Data are mean ± SEM; *p < 0.05.

## Data Availability

The data that support the findings of this study are available on request from the corresponding author. The data are not publicly available due to privacy or ethical restrictions.
