# Supplementary material for: Resveratrol Attenuates Obesity by Inducing GDF15 Expression via the p38‐ATF3 Signaling Pathway
Source: Food Sci Nutr. 2026 Jun 28;14(7):e72052. doi: 10.1002/fsn3.72052 (PMC13311194; doi:10.1002/fsn3.72052)
Supplement: Supplementary file 1 — Figure S1: Validation of Atf3 knockdown in MEF cells. QPCR analysis demonstrates that transfection with Atf3‐specific siRNA effectively reduces endogenous Atf3 mRNA expression. Data are mean ± SEM; *p < 0.05. Figure S2: Quantification of protein expression in HepG2 cells. Quantification of ATF3 (A) and GDF15 (B) protein expression in HepG2 cells. Data are mean ± SEM; *p < 0.05. Figure S3: Resveratrol effects in mice on standard chow diet. (A) Average daily food intake in mice fed standard chow with or without resveratrol for 1 week. (B) Serum GDF15 levels after resveratrol treatment. Data are mean ± SEM; *p < 0.05. [file FSN3-14-e72052-s001.docx]

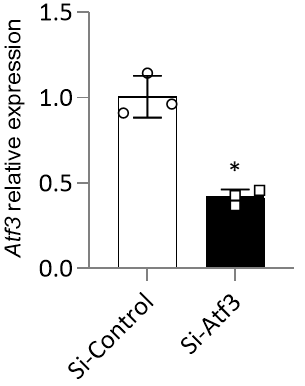


**Figure S1. Validation of Atf3 knockdown in MEF cells.**

QPCR analysis demonstrates that transfection with Atf3-specific siRNA effectively reduces endogenous Atf3 mRNA expression. Data are mean ± SEM; *p < 0.05.


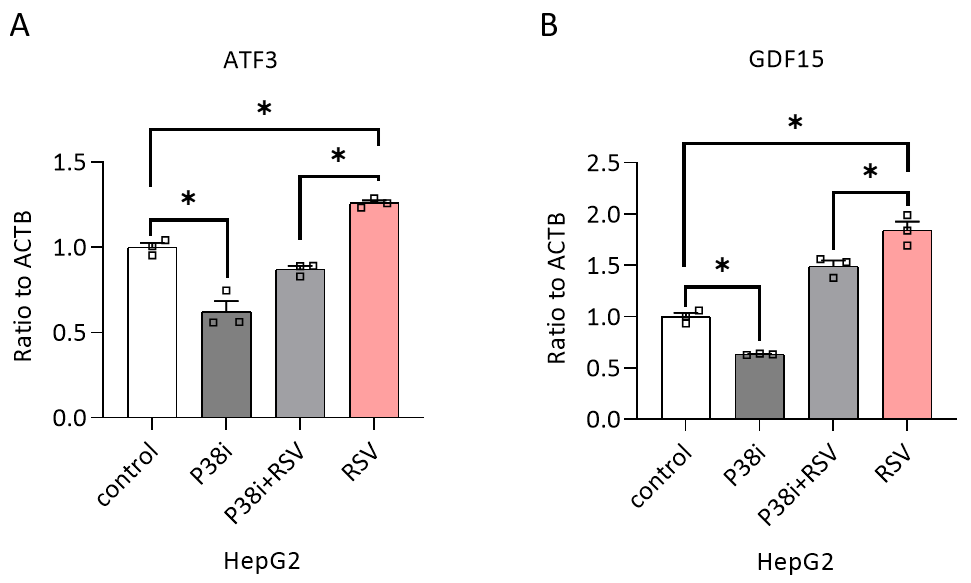


**Figure S2. Quantification of protein expression in HepG2 cells.**

Quantification of ATF3 (A) and GDF15 (B) protein expression in HepG2 cells. Data are mean ± SEM; *p < 0.05.


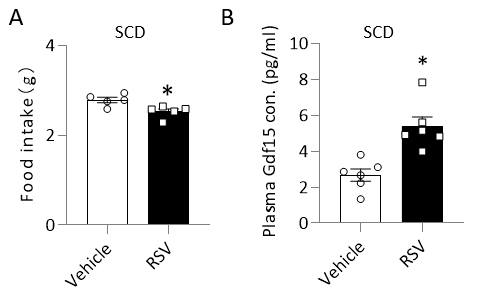


**Figure S3. Resveratrol effects in mice on standard chow diet.**

(A) Average daily food intake in mice fed standard chow with or without resveratrol for 1 week. (B) Serum GDF15 levels after resveratrol treatment. Data are mean ± SEM; *p < 0.05.
